# Effects of customized CAD/CAM abutments on cytokine levels in peri-implant crevicular fluid during early implant healing: a pilot study

**DOI:** 10.1007/s00784-022-04826-x

**Published:** 2022-12-24

**Authors:** Christian Wehner, Gabor Fürst, Tom Vaskovich, Oleh Andrukhov, Christoph Vasak, Andreas Moritz, Xiaohui Rausch-Fan

**Affiliations:** 1 Division of Conservative Dentistry and Periodontology, University Clinic of Dentistry, Medical University of Vienna, Vienna, Austria; 2 Dental Technician Laboratory, University Clinic of Dentistry, Medical University of Vienna, Vienna, Austria; 3 Competence Center for Periodontal Research, University Clinic of Dentistry, Medical University of Vienna, Vienna, Austria; 4 Division of Oral Surgery, University Clinic of Dentistry, Medical University of Vienna, Vienna, Austria

**Keywords:** Dental implant-abutment design, Cytokines, Bone implant interface, Osseointegration

## Abstract

**Objectives:**

This study aimed to assess levels of biomarkers associated with inflammation and tissue destruction in peri-implant crevicular fluid (PICF) of implants provided with customized or standard healing abutments during early implant healing.

**Materials and methods:**

Thirty implants were placed in 22 patients with partial posterior edentulism. Subsequently, test group implants (*n*=15) received one-piece titanium abutments that were fabricated using computer-aided design/computer-aided manufacturing (CAD/CAM). Control group implants (*n*=15) were provided with standard abutments. PICF collection and standardized periapical radiographs were carried out at suture removal one week later, following crown delivery after 3 months and at 6 months. Expression of C-reactive protein (CRP), interferon-γ, tumor necrosis factor (TNF)-α, interleukin (IL)-1α, IL-1β, IL-2, IL-4, IL-6, IL-8, IL-10, IL-12A, IL-17A, macrophage inflammatory protein (MIP)-1α, matrix metalloproteinase (MMP)-13, osteopontin, osteoactivin, Receptor Activator of NF-κB (RANK), and TGF-β were analyzed using a multiplex ELISA kit.

**Results:**

Both groups showed a significant decrease in protein expression of CRP, IL-1β, IL-6, IL-8, MIP-1α, osteopontin, osteoactivin, and TGF-β, while MMP-13 levels increased during the observation period. A rise in OPG and RANK levels was detected among customized abutments. Expression of CRP was higher, whereas IL-1β, IL-1α, and MIP-1α were decreased in control compared to test group implants after 6 months. Marginal bone loss did not depend on abutment modality.

**Conclusions:**

Both abutment types showed distinctive temporal expression of inflammatory biomarkers during 6 months following implant placement.

**Trial registration:**

ISRCTN98477184, registration date 18/05/2022

**Clinical relevance:**

Customized healing abutments exert similar effects on inflammation during early implant healing compared to standard healing abutments.

## Introduction

The function and integrity of the peri-implant soft tissue seal play a key role in implant healing by ensuring healthy conditions of the transmucosal area, thereby paving the way for stable osseointegration [1]. The soft tissue seal serves as a protective barrier separating the oral environment from the underlying peri-implant bone and consists of an epithelial and a connective tissue zone [2, 3]. Depending on the prosthetic protocol for non-submerged dental implants, the healing abutment has to be removed and reconnected several times between implant placement and the delivery of the final restoration. As each disconnection of restorative components involves a disruption of the soft tissue adhesion, it has been suggested that repeated manipulations might negatively impact peri-implant soft- and hard-tissue conditions [4–6]. In this context, some studies showed adverse effects of the disconnections on marginal bone levels surrounding the implant.

Early investigations by Abrahamsson et al. demonstrated in a pre-clinical study that increasing the number of abutment removals and reconnections up to five times compromised the mucosal barrier, leading to the epithelium’s apical migration and increasing marginal bone resorption [7]. These findings were later confirmed by studies demonstrating that standard prosthetic protocols for implant treatment requiring frequent abutment changes were associated with a decreased subepithelial connective tissue attachment in dogs [5] and with an increased marginal bone loss during the implant healing period in a clinical trial [8]. More specifically, a meta-analysis reported a weighted mean difference in crestal bone loss of 0.19 mm (95% confidence interval, 0.06–0.32 mm) in favor of final abutment placement after implant insertion compared to multiple abutment manipulations [4]. Consequently, it has been suggested that minimizing the number of abutment dis- and reconnections could have beneficial effects on marginal bone levels [9].

Another approach reflecting the tissue conditions surrounding an implant is to analyze the peri-implant crevicular fluid (PICF) [10]. In line with the abovementioned findings on interproximal bone level, repeated abutment removal and reconnection of more than three times were associated with higher IL-1β levels in PICF compared to implants that received final abutments at implant uncovery, suggesting promotion of inflammation [11].

As a result of these studies, prosthetic protocols that aim to reduce tissue trauma following implant insertion have been developed. According to the “one abutment at one time” protocol, the definitive restorative abutment is placed at the time of implant insertion to prevent removal during the healing phase [12]. Moreover, in addition to reducing the number of abutment manipulations, customized abutments can be provided through a virtual design based on computer-guided planning in order to simulate the natural anatomy of the prosthetic crown [13]. To manufacture a customized healing abutment after implant insertion, one digital impression is required which can also be used for the definitive crown design. After the healing period, the abutment is removed in order to attach the crown based on the emergence profile of the abutment. In contrast, conventional impression taking takes at least two abutment manipulations prior to definitive restoration.

However, the influence of minimizing the number of dis- and reconnections of the healing abutment combined with abutment customization on the underlying processes of early implant healing is poorly understood. This pilot study aimed to assess the effects of one-piece customized titanium abutments on a broad range of biomarkers associated with inflammation and tissue degradation in the peri-implant crevicular fluid (PICF) as well as the marginal bone loss observed during the early healing phase. We hypothesized that the use of customized healing abutments induces a reduced inflammatory response compared to standard healing abutments.

## Materials and methods

### Study population and design

**Fig. 1:**
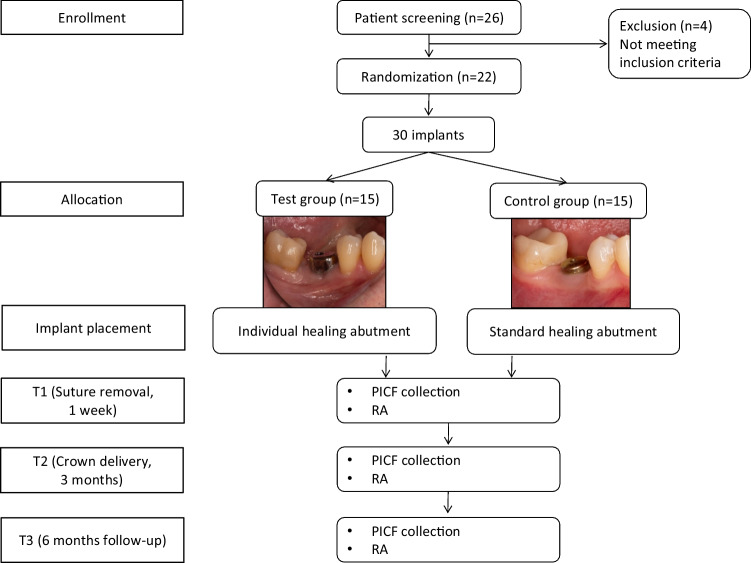
Flow chart of the study design and clinical procedures. PICF, peri-implant crevicular fluid; RA, radiographic assessment

In this prospective study, participants were recruited at the University Clinic of Dentistry, Vienna, between January 2019 and July 2021. Patients were included after obtaining their written consent and if the following inclusion criteria were met: (1) >18 years old, (2) one or more missing tooth/teeth in the molar region of the upper and/or lower jaw, (3) adequate bone quality and availability for implant placement, (4) no signs of inflammation in the region where implant placement is planned, (5) good systemic health conditions, (6) stable occlusion, and (7) willing to participate and attend follow-up appointments. Patients were excluded in case of the following criteria: the presence of untreated periodontitis, smokers (> 10 cigarettes per day), alcoholism or drug abuse, history of chemotherapy or radiation, and diabetes with > 7.5 HbA1c. A total of 30 titanium implants (C1, MIS Implants Technologies, Bar Lev Industrial Park, Israel) were allocated to two groups using online available randomization tools (https://www.randomizer.org/). The general study design is summarized in Fig. 1. The study protocol was approved by the Ethics Committee of the Medical University of Vienna (EK-Nr. 1807/2017) and performed in accordance with the Helsinki Declaration of 1975, as revised in 2013, and the “Good Scientific Practice” guidelines of the Medical University of Vienna. The trial was registered at ISRCTN registry (https://doi.org/10.1186/ISRCTN98477184).

### Surgical and prosthetic protocol

Prior to surgery, randomization was performed at each implant site to determine if the implant will be provided with a customized or a standard healing abutment. All patients received 2 g amoxicillin one hour before surgery as a standard clinical protocol [14]. Surgery was performed under local anesthesia using 1–2 cartridges of articaine with 1:100000 epinephrine (Ultracain D-S forte, Sanofi, France) for each implant site. Following a crestal incision and extension to the buccal and lingual aspects of adjacent teeth, a full-thickness flap was elevated, and site preparation was performed according to the manufacturer’s instruction.

**Fig. 2:**
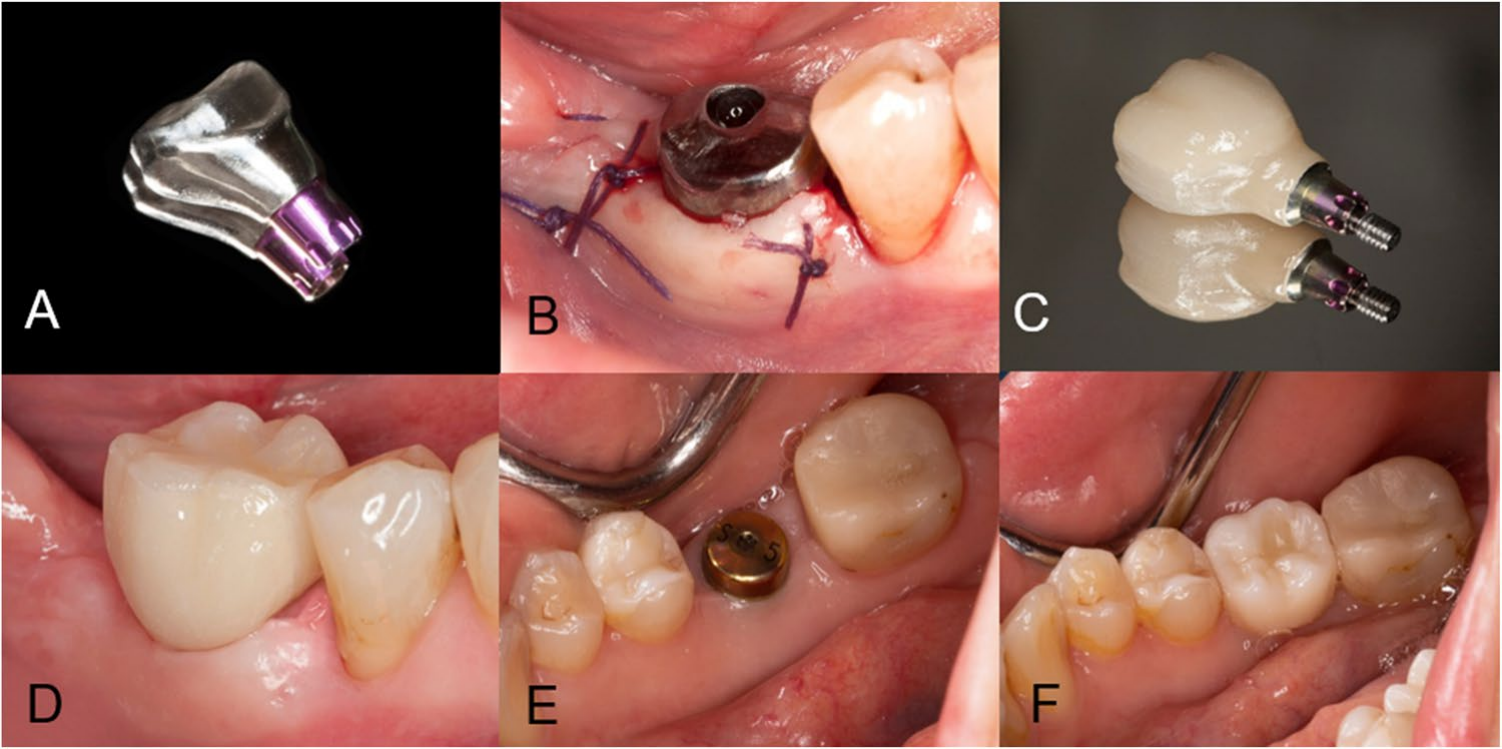
Case report for the test and control group. Manufacturing of a CAD/CAM-based customized one-piece titanium abutment (**A**) and fixation following implant placement (**B**). Delivery of definitive crown (**C**) after 3 months (**D**). Control group implants were provided with standard healing abutments (**E**) and definitive crown (**F**)

After implant insertion in the test group participants, digital impressions (TRIOS 3, 3shape, Denmark) were taken to manufacture a customized healing abutment from titanium blanks (Ti-blank, MIS Implants Technologies, Bar Lev Industrial Park, Israel) using dental modeling software (Ceramill Mind 3.0, Amann Girrbach, Germany) and a 5-axis milling unit (Ceramill Motion 2*,* Amann Girrbach, Germany). Customized abutments were subsequently delivered to the patient and installed. In the control group, concave titanium standard healing abutments were installed after surgery. Mucoperiosteal flaps were adapted to the healing abutments and sutured (5-0 coated vicryl, Ethicon, US). Suture removal was performed in all patients one week after implant placement (T1). Three months following surgery, the study participants received a screw-retained crown made out of zirconia (T2). In the control group, conventional impression taking (Impregum Penta Soft Polyether, 3M, US) was performed, and patients received the definitive crown 7 days later. Test group participants were provided with a crown bonded to a titanium base harboring the matching shape of the previous customized healing abutment; the aim was to minimize tissue trauma. Six months after surgery, patients were scheduled for a follow-up visit (T3). Figure 2 shows the installation of a customized abutment followed by crown delivery after 3 months.

### PICF collection

PICF sample collection was performed on each visit at 4 sites (mesio-buccal*,* disto-buccal, mesio-lingual, disto-lingual) of each implant. After gentle air drying and isolation of the gingiva with sterile gauze to prevent saliva contamination, a sterilized paper collection strip (PerioPaper strips, Oraflow Inc., Plainview, NY, USA) was inserted into the peri-implant sulcus until slight resistance was reached for 30 s. If contamination with blood was observed, the samples were discarded. The adsorbed volume was determined using a calibrated electronic volume quantification unit (Periotron 8000, Oralflow Inc., Plainview, NY, USA). The four strips of each implant were pooled together in Eppendorf tubes and subsequently stored at −80°C until further processing. Before analysis, the samples were unfrozen, and protein extraction following an elution method was performed as described previously [15]. Twenty microliters of extraction buffer (24.5mL phosphate-buffered saline (pH 7.4), 125ml phenylmethylsulfonylfluoride (PMSF; Sigma Chemical, St. Louis, MO), 200mM in methanol, 1 mg/ml in water, and 83.5 ml of 30% human serum albumin (Sigma Chemical, St. Louis, MO)) were pipetted onto the cellulose part paper collection strip. The strips were put inside Eppendorf tubes and centrifuged at 2000 rpm at 4° C for 5 min. To gain a total volume of 100 μl for each tube, this step was replicated four additional times. The entire product was then stored on dry ice for subsequent analysis of biomarker concentration.

### Cytokine analysis

A commercial human multiplex ELISA kit (Quantibody Human Periodontal Disease Array 1 Kit, RayBiotech, Norcross, Georgia, USA) was used to assess the expression of C-reactive protein (CRP), interferon (IFN)-γ, tumor necrosis factor (TNF)-α, transforming growth factor (TGF)-β, interleukin (IL)-1α, IL-1β, IL-2, IL-4, IL-6, IL-8, IL-10, IL-12A, IL-17A, macrophage inflammatory protein (MIP)*-*1α*,* matrix metalloproteinase *(*MMP)-9, MMP-13, osteopontin, osteoactivin, osteoprotegerin, and Receptor Activator of NF-κB (RANK). Concentrations were determined by generating a standard curve for comparison.

### Radiographic assessments

Radiographic examinations using standard parallel technique, perpendicular to the long axis of the implants, were conducted after implant placement (T1) as well as at the 3- and 6-month follow-up visit (T2 and T3, respectively). The distances on the radiograph were calibrated using the known implant diameters. All radiographs were assessed by a blinded independent examiner who was not involved into implant placement or the follow-up appointments (XR). Marginal bone loss (MBL) was determined as the distance from the implant shoulder and the interproximal bone level as described previously [16]. Assessments were performed on the mesial and distal aspect of the implant; they were presented as the mean of the two values for each respective time point.

### Statistical analysis

The statistical analysis for all quantitative variables was conducted using SPSS Statistics (IBM, Armonk, NY). The Wilcoxon Signed Rank test was performed to compare the levels of single parameter in PICF samples between different time points. The Mann*-*Whitney *U-*test was used to compare the test and control group regarding the levels of cytokine concentration. Quantitative data are expressed and displayed as mean ± SD. *P* values <0.05 were considered to be statistically significant.

## Results

Table 1 shows the demographic and clinical characteristics of the study participants. A total of 30 implants were placed in 22 patients (15 females). Two patients received both test and control group implants. One of these patients was provided with two test and two control group implants. Another patient received two test group implants and one participant was provided with three control group implants. The mean age at the time of implant placement was 47.7 vs. 48.1 years (range 27.1–58.5 vs. 28.9–66.7 years) for the test and control groups, respectively. Five study participants of the test group were smokers (≤10 cigarettes/day), compared to eight in the control group. Implants were most often placed in location 46 (*n*=6, 40%) in the test group, while location 36 (*n*=4, 26.7%) was most common in the control group.

**Table 1:** Demographic and clinical characteristics for the test and control groups

Test group (*n*=15)			Control group (*n*=15)		
Age (mean ± SD [min; max])	47.7 ± 9.1 [27.1-58.5]		Age (mean ± SD [min; max])	48.1 ± 11.5 [28.9-66.7]	
Gender (female; *n* [%])	7 [46.7]		Gender (female; *n* [%])	8 [53.3]	
Smoking status (smoker; *n* [%])	5 [33.3]		Smoking status (smoker; n [%])	3 [20]	
Implant location	*N*	%	Implant location	*N*	%
16	0	0	16	3	20
17	0	0	17	0	0
26	2	13.3	26	2	13.3
27	1	6.7	27	2	13.3
36	4	26.7	36	1	6.7
37	1	6.7	37	1	6.7
46	6	40	46	4	26.7
47	1	6.7	47	2	13.3

### Bone level alterations

The results for the marginal bone level (MBL) alterations are presented in Table 2. MBL values significantly decreased in both test and control group at 3-month follow-up (0.24 ± 0.09 mm and 0.23 ± 0.14 mm, respectively, *P* < 0.01) and at the 6-month follow-up (mean 0.38 ± 0.1mm and 0.33mm ± 0.14 mm, respectively, *P*<0.01) compared to T1. No significant difference was found between the test and control groups at any milestone.

**Table 2:** Marginal bone level (MBL) alterations at 3 and 6 months (T2 and T3)

		Loading (3 months)	6 months
Mean ± *SD*	Test group	0,24 ± 0,09	0,39 ± 0,1
	Control group	0,23 ± 0,14	0,33 ± 0,14
*p*-value	Test group	< 0.01	< 0.01
	Control group	< 0.01	< 0.01

### PICF biomarker changes

**Fig. 3:**
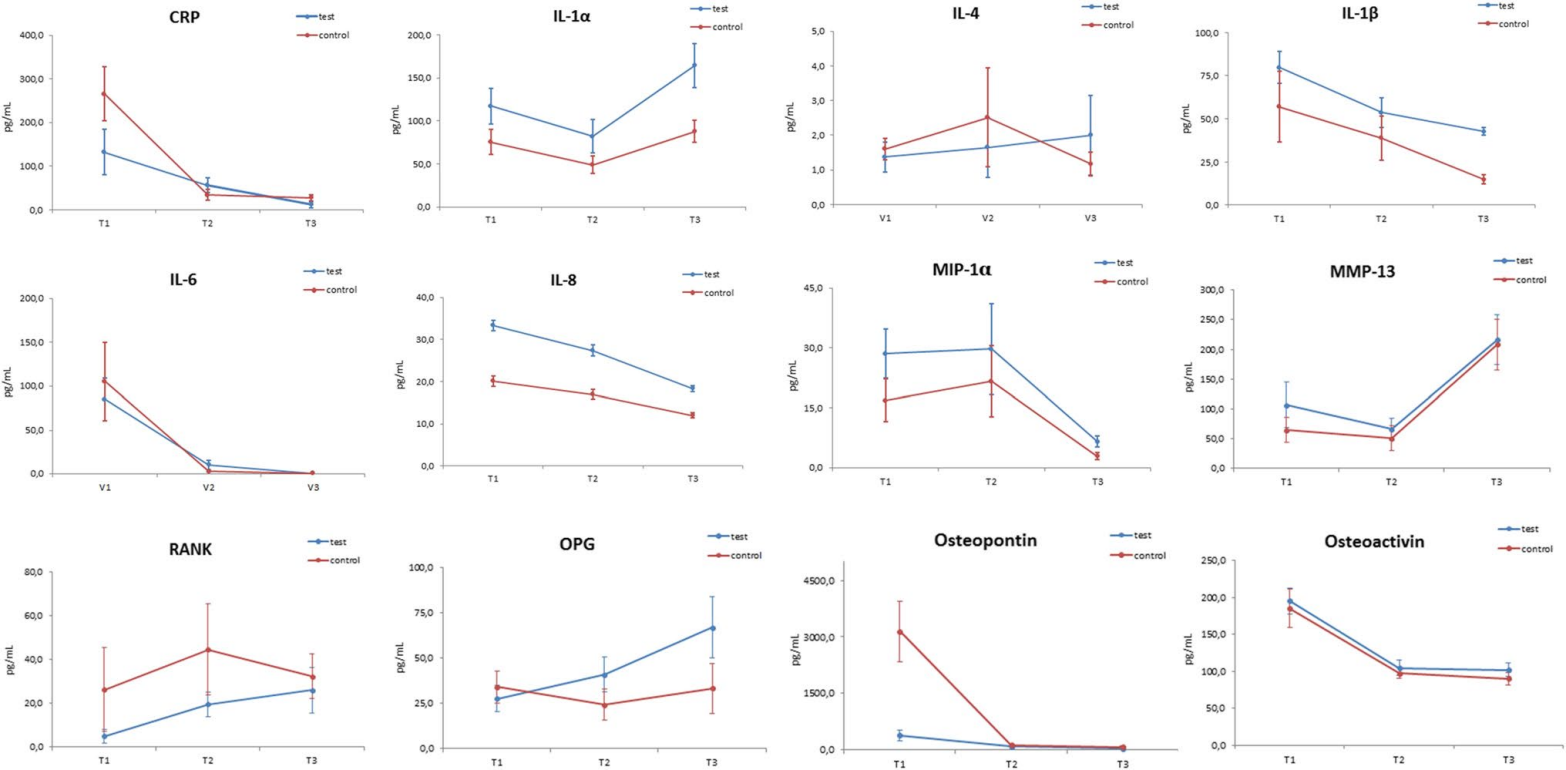
Changes of PICF biomarker levels in the course of the study at T1, T2, and T3

The levels in the course of the study of both the test and control groups are presented in Fig. 3. IL-17, IL-12, IL-10, IL-2, IFN-γ, TGF-β, and TNF-α were below the detection limit in the majority of samples, and therefore, these parameters were not included in the analysis. In all samples, MMP-9 was measured above the maximum detection limit; therefore, data were not presented.

The levels of CRP, IL-6, MIP*-*1α, osteopontin, and osteoactivin gradually decreased at T3 compared to the assessment at suture removal (T1) for the test and control groups. CRP levels of the test group were also lower at T3 than at T2 and decreased in the control group from T1 to T2. IL-6 showed a reduction in the test group from T1 to T2 and from T2 to T3. Also, in the control group, IL-6 levels decreased from T1 to T2. MIP*-*1α exhibited lower levels at T3 compared to T2 in the test and control groups. IL-8 expression was decreased in the test group at T3 compared to T1. In the control group, IL-1β and TGF-β showed a significantly lower detection level at T3 compared to T1. Osteopontin also decreased in the test group from T1 to T2 and from T2 to T3. Osteoactivin levels diminished in the test and control groups from T1 to T2.

An increase in concentration was observed for IL-1α levels from T2 to T3 in the control group. Also, OPG and RANK levels in the test group were higher at T3 compared to T1. MMP-13 levels increased in the test and control groups from T2 to T3 and in the control group as well from T1 to T3.

No changes in the levels of IFN-γ, IL-2, IL-4, IL-10, IL-12, IL-17, and TNF-α in both groups were observed during the study.

Some differences in the investigated parameters were observed between the test and control groups. At T3, the PICF levels of CRP were superior in the control group than in the test one. Also, IL-1α, IL-1β, and MIP*-*1α were increased in the test group compared to the control at T3.

## Discussion

The results of the present pilot study revealed fading soft tissue inflammation in both treatment groups as well as similar bone remodeling during early implant healing. Some significant intergroup differences in PICF were detected. At 6 months, CRP was less expressed in PICF of the test group than of the control group. CRP is produced in the liver and delivered to the sulcus through saliva and or blood vessels. As blood levels of CRP were not measured in the present study participants, it cannot be ruled out that increased CRP levels could also be due to increased systemic levels in the control group. Furthermore, IL-1β, IL-1α, and MIP*-*1α were detected at higher levels in the test group compared to the control. IL-1β and IL-1α are pro-inflammatory proteins that are increased in response to growth factors and pro-inflammatory or stress-associated stimuli [17]. In contrast to CRP, production of IL-1β, IL-1α, and MIP*-*1α takes place locally in the sulcus fluid by macrophages or epithelial cells; this indicates a local enhancement of the inflammation process in PICF of implants provided with customized abutments.

In both abutment groups, a significant decrease in the expression of CRP, IL-6, MIP*-*1α, osteopontin, and osteoactivin in PICF during the 6-month observation period was measured; this suggests a reduction of the inflammation process. While CRP is known as a marker for systemic inflammation, it has also been detected in PICF at peri-implantitis sites [18]. As a classical pro-inflammatory cytokine, IL-6 has been used to assess peri-implant inflammation in PICF [19]. MIP-1α/CCL3 is secreted by macrophages and plays a role in chemotaxis and stimulation of cell migration during inflammation and bone resorption. It has also been hypothesized to have a potential as a diagnostic tool for peri-implant tissue conditions, although scientific evidence is inconsistent [20, 21]. As a glycosylated phosphoprotein, osteopontin is expressed by both osteoblasts and osteoclasts and is involved in bone resorption and remodeling as well as inflammatory processes [22]. Osteoactivin enhances osteoblast differentiation during matrix maturation and mineralization in osteoblast progenitor cells [23]; however, it has also been associated with inflammation [24].

In contrast to the abovementioned cytokines, both groups showed an increase in the MMP-13 levels in PICF at 6 months compared to suture removal. MMP-13 plays a key role in regulating wound granulation tissue growth and is involved in the expression of genes associated with inflammation, proteolysis, and cell viability [25]. A promotion of MMP-13, therefore, might correlate with wound healing progression during the observation time.

In the test group, an increase in OPG and RANK has been observed after 6 months compared to suture removal following implant placement. OPG and RANK are part of the RANK/RANKL/OPG system, thereby triggering bone metabolism. RANK is an osteoclast-bound receptor, which is activated by its ligand RANKL, resulting in osteoclast differentiation [26]. The present findings suggest an increase in bone turnover in the test group; however, it has to be considered that RANKL was not assessed in the array setting used.

Limited marginal bone loss within the first years following implant placement has been considered an adaptation to surgical trauma and implant loading [27]. No significant differences in the marginal bone levels between the two abutment groups could be identified during the observation period of the present study. Our findings are in line with a study by Moreira et al., who compared the placement of a definitive abutment after implant placement to three times disconnection and reconnection of the healing abutment; they reported a slightly inferior bone loss at 6 months of 0.14 ± 0.18 mm and 0.23 ± 0.29, respectively [28]. However, the present study focused on early implant healing, and possible differences in peri-implant bone level might be detected at a later follow-up visit.

When discussing the present results, it has to be taken into account that other factors in addition to abutment design might also contribute to the difference in cytokine expression between the two abutment groups. In vitro studies have shown that surface material also determines the susceptibility of gingival fibroblasts toward inflammatory stimuli [29]. In line with these findings, the expression of inflammatory cytokines in PICF in clinical settings has been influenced by abutment material [30]. Moreover, also the release of titanium particles during tribocorrosion as a result of material degradation might enhance inflammation. Thus, assessing the specific effects of each abutment modality on the inflammatory process would need further investigation.

This pilot study displays a certain number of limitations. The focus of the present investigation was limited to early implant healing and long-term effects were not assessed. Thus, there is a need for future studies to evaluate the impact of customized one-piece abutments on the healing process over the long-term. Although a broad cytokine profile was assessed in this study, complexity of the inflammation process could only partly be displayed. Also, other aspects essential to the healing process such as angiogenesis, proliferation, or host response could be subject of further studies.

Taken together, customized abutments represent an alternative to standard healing abutments; however, higher production costs and treatment time have to be considered.

## Conclusion

Within the limits of this study, we did not observe substantial differences between customized and standard healing abutments with regard to inflammatory markers and marginal bone levels. Subsequently, both treatment protocols can be equally recommended.

## Data Availability

Data are available on request from the authors.
